# Use of Anti-VEGF Agents in Glaucoma Surgery

**DOI:** 10.1155/2017/1645269

**Published:** 2017-06-27

**Authors:** Mark Slabaugh, Sarwat Salim

**Affiliations:** ^1^Department of Ophthalmology, William H. Havener Eye Institute, The Ohio State University Medical Center, Columbus, OH, USA; ^2^Department of Ophthalmology, Medical College of Wisconsin, 925 N. 87th Street, Milwaukee, WI 53226, USA

## Abstract

A number of antivascular endothelial growth factor agents are currently available to treat various ocular conditions. These agents have similar, but distinct, biologic qualities and have been explored in the management of neovascular glaucoma and in glaucoma surgery. Several different delivery methods are described, and because these medications are routinely given as intraocular injections, some benefits over traditional antifibrotic medications when used in glaucoma surgery are noted. These agents effectively induce regression of anterior segment neovascularization and facilitate initial surgical management of neovascular glaucoma, but the long-term outcome of this condition remains dependent on definitive management of the underlying process. Use in trabeculectomy or tube shunt procedures for other types of glaucoma has shown promise in modulating bleb morphology but has not yet been found to be as effective as traditional antifibrotic agents. There are reports of persistently raised intraocular pressure after repeated use of the anti-VEGF agents, possibly related to frequency of injection. These medications have wide application in the field of surgical glaucoma, but a definitive role has yet to be defined.

## 1. Introduction

The potential of antivascular endothelial growth factor (anti-VEGF) agents to modify the disease course of neovascular glaucoma (NVG) was recognized shortly after their use in the treatment of age-related macular degeneration was reported. These medications were noted to induce rapid regression of the anterior segment neovascularization that characterizes NVG. This response has changed the way that newly diagnosed NVG is managed, although the effect of these agents on long-term clinical outcomes is less clear. Due to the role of VEGF in fibrosis, the anti-VEGF agents have been widely used not only in NVG but also to modify the wound healing response in traditional glaucoma surgery. Experiments have been performed considering the effect on bleb morphology, trabecular function, and retinal ganglion cell survival. We review the current use of these agents in various surgical glaucoma scenarios, as well as the direct effects of their injection on intraocular pressure (IOP).

## 2. Anti-VEGF Agents

The effect of VEGF on wound healing is related to its role in both vascularization and fibrosis of tissues involved. Each of the VEGF isoforms is active in normal and pathologic vascular endothelial growth and permeability. These growth factors have been found to affect fibrosis and collagen deposition in normal wound healing [1]. Due to this dual mechanism of action, the ability to block this family of molecules has the potential to impact diseases where there is pathologic overexpression of VEGF or when it is desirable to modulate the normal healing response, as in glaucoma filtering surgery.

Each of the anti-VEGF agents that are currently used to treat ocular conditions has been applied specifically in glaucoma management and surgery. Pegaptanib (Macugen, Pfizer, New York), bevacizumab (Avastin, Genentech Inc., San Francisco, CA), ranibizumab (Lucentis, Genetech Inc., San Francisco, CA), and aflibercept (Eylea, Regeneron, New York) vary in their affinity for the subtypes of the VEGF molecule.

Pegaptanib is an aptamer that selectively prevents the VEGF_165_ isomer of VEGF-A from binding to its receptors. Bevacizumab is a full-sized monoclonal antibody that binds all isomers of VEGF-A. Ranibizumab is the antigen-binding fragment (Fab) of a similar antibody that has a slightly higher affinity for all isomers of VEGF-A. Aflibercept is a recombinant fusion protein that also binds all isomers of VEGF-A and also binds VEGF-B and the related placental growth factor (PlGF) [2].

Each of these agents has been described as efficacious in the initial management of NVG [3–6] and has been used as an experimental surgical adjunct in traditional glaucoma filtering surgeries [1, 7–9]. There is extensive literature addressing the differential clinical response of each medication in the management of retinal diseases, but there are not currently comparative studies in either the management of NVG or as agents for use in glaucoma surgery. For the purposes of this review, the different agents will be highlighted as they have been reported, but it should be noted that there is no current evidence to suggest one is more efficacious than another in managing NVG or filtering glaucoma surgery. It is also anticipated that biosimilar drugs to each of these agents will become available. The most widely reported one, Razumab (Intas Pharmaceuticals, Ahmedabad, India), is a biosimilar to ranibizumab and is approved for use in India. Limited data is currently available on its role in NVG or glaucoma filtering surgery.

## 3. Delivery Methods Addressing NVG

For the management of NVG, the initial delivery of the anti-VEGF agents is generally in a standard intravitreal injection, although other methods have been described. Waisbourd et al. described some success inducing regression of neovascularization following four times daily topical administration of bevacizumab [10]. Several authors have reported rapid regression of anterior segment neovascularization following injection of bevacizumab into the anterior chamber [11–13]. A complicating factor to the standard intravitreal injection is that patients who present with a new diagnosis of NVG often have very elevated IOP, and the additional intravitreal volume exacerbates this situation. Patients with elevated IOP are frequently managed medically in clinic until the IOP reaches a more acceptable level. Alternatively, an anterior chamber paracentesis can be performed at the time of the intravitreal injection. In this setting, there may also be a role for these alternative methods of delivery.

## 4. Delivery Methods in Filtering Surgery

Use of antifibrotic agents such as 5-fluorouracil (5-FU) and mitomycin C (MMC) during traditional glaucoma surgery has been confined primarily to either saturated sponges at the time of surgery or subconjunctival injection during or after surgery. These routes of administration are used due to their immediate and local effect and, more importantly, to limit their toxicity to intraocular structures. The anti-VEGF agents are considered safe for intraocular injection, and coincidentally, investigators have used injections into the vitreous and anterior segment, as well as the traditional modes of topical and subconjunctival administration.

Nomoto et al. demonstrated in a rabbit model that intravitreal administration resulted in a higher concentration of bevacizumab within the eye compared to a subconjunctival administration; however, this has limited application to glaucoma surgery as in this case the site of desired action is extraocular [14]. Moisseiev et al. observed intravitreal concentrations of bevacizumab in patients undergoing vitrectomy and showed that, in patients without prior vitrectomy, the half-life was approximately 5 days [15]. They also showed that, in one patient who had undergone a prior vitrectomy, the calculated half-life was greatly reduced to less than 1 day.

The application of these findings to both the treatment of NVG and the management of glaucoma filtering surgery is not yet completely understood. In general, when a rapid effect is needed to control anterior segment neovascularization, intravitreal or intracameral injection of an anti-VEGF agent will be effective. Given the shorter half-life concentrations observed in postvitrectomy eyes, faster recurrence of anterior segment neovascularization might be expected in the absence of more definitive therapy such as panretinal photocoagulation (PRP) when anti-VEGF agents are injected into either the anterior chamber or a postvitrectomy posterior chamber. It is possible that a similar effect will become pertinent in the use of anti-VEGF agents in glaucoma filtering surgery. Direct application by sponges or injection will result in much higher local concentrations initially; however, a more prolonged low-level effect could be observed following injection into the vitreous. The clinical relevance of these effects is currently unknown.

## 5. Indications and Outcomes in Glaucoma Surgery for NVG

Neovascular glaucoma occurs when an ischemic process induces secondary angle closure due to fibrovascular proliferation on the iris and into the anterior chamber angle. The most common causes include diabetic retinopathy, retinal vein occlusions, and ocular ischemic syndrome. Historically, outcomes have been poor, as glaucoma filtering surgeries have a high rate of failure and recurrence of the condition is common [16]. Even with adequate control of the underlying condition, visual outcomes have been poor. Control of elevated IOP often requires multiple interventions including medications, tube shunt surgery, and cyclodestructive procedures.

With the advent of the anti-VEGF agents, the success in controlling active neovascularization has been greatly enhanced. Several authors have reported positive short- and long-term results of various injection regimens in NVG with the various anti-VEGF medications [6, 11–13, 17]. With adequate control of the neovascularization, glaucoma surgery has become possible, although it still depends on control of the underlying condition. The long-term success of trabeculectomy in NVG has not been definitively shown to be improved by injecting bevacizumab as compared to MMC alone, although changes in bleb vascularity have been reported [18, 19]. Several authors have also reported similar results with glaucoma tube shunt implantation in NVG. Although there are beneficial effects including less anterior segment bleeding, the long-term outcomes with regard to visual acuity and IOP control are more dependent on definitive control of the underlying condition than on any specific perioperative anti-VEGF injections [20–22].

Laser therapy is often employed in the management of NVG. Panretinal photocoagulation (PRP) may be performed to address the underlying retinal disease and to reduce retinal oxygen demand and release of VEGF prior to surgical intervention for NVG. In a retrospective, consecutive case-control study, Ehlers et al. compared combined PRP and intravitreal bevacizumab to PRP monotherapy [23]. The authors reported that combination treatment resulted in more rapid decrease in IOP and increased frequency and faster regression of neovascularization. Cyclophotocoagulation (CPC) diode laser is often reserved for eyes with poor visual potential or when other surgical options have failed. CPC laser is effective, quick, and useful for patients who are unable to undergo incisional surgery. Gosh et al. evaluated combined CPC and intravitreal bevacizumab in eyes with NVG and reported rapid regression of neovascularization, IOP control, and symptomatic relief [24].

## 6. Indications and Outcomes in Glaucoma Surgery Aside from NVG

The role of antifibrotic agents in traditional glaucoma filtering surgery is well known. With the use of 5-FU and MMC, trabeculectomy in particular became more successful at reaching target IOP [25]. With the increase in efficacy, however, there has also been an increase in the incidence of bleb-related complications. Consequently, there is great interest in using a more focused approach to wound healing modulation. VEGF has several roles in wound healing, and the previously mentioned differential affinity of the different anti-VEGF agents may someday be used to exploit this. VEGF_165_ and VEGF_121_ more directly affect angiogenesis while the isomer VEGF_189_ has more of an impact on fibrosis [8].

Multiple studies have evaluated the use of bevacizumab or ranibizumab as an alternative or adjunct to MMC at the time of trabeculectomy [26–30]. As mentioned above, routes of administration include topical, subconjunctival, intracameral, and intravitreal [31]. Many of the reports have shown a difference in bleb morphology in eyes treated with anti-VEGF agents. Early postoperative results have shown less vascular and more diffuse blebs compared to MMC; however, this effect appears to fade and longer-term outcomes show more vascularity and higher IOP when they were used as single agents compared with MMC [26, 29, 32].

There are also reports of subconjunctival injection of bevacizumab for use in rescuing failing filtering blebs [33, 34]. The use of 5-FU in particular is well described for this, and Freiberg et al. showed a decrease in the number of 5-FU injections required when used in conjunction with bevacizumab [33]. In a similar pattern of results to those evaluating stand-alone use in trabeculectomy, bleb vascularity was reportedly improved following these treatments; however, they did not demonstrate an improvement in long-term IOP. Postoperative use of anti-VEGF agents may have benefit in bleb rescue, but studies have not demonstrated a significant effect on IOP to date.

In glaucoma tube shunt surgery, the use of antifibrotic agents is less well defined than in trabeculectomy. Several large studies have addressed the use of MMC during tube shunt implantation and have failed to show a long-term effect on IOP [35, 36]. There are, however, newer reports of a limited role using MMC and 5-FU in tube shunt surgery [37]. Many studies have evaluated the use of anti-VEGF agents as adjuncts in tube shunt surgery for NVG, but only a few small studies have examined their use for the same procedures in other forms of glaucoma [38, 39]. Rojo-Arnao et al., using a postoperative series of bevacizumab injections, showed a decreased hypertensive phase without a long-term impact on IOP [38]. Desai et al. used intravitreal ranibizumab at the time of tube implantation surgery and found a trend toward improved outcome [39].

There have been some reports of rapid conjunctival dehiscence or necrosis with the subconjunctival injection of bevacizumab or ranibizumab. Georgalas et al. reported a case of conjunctival necrosis that occurred in a patient with a long-standing trabeculectomy shortly after a ranibizumab injection for AMD [40]. A prospective trial by Sengupta et al. comparing bevacizumab with either sponges or injection to MMC sponges used at the time of filtering surgery documented a case of conjunctival necrosis in the bevacizumab injection group [41]. Finally, Miraftabi and Nilforushan reported two cases of severe conjunctival dehiscence following placement of a glaucoma drainage implant with intraoperative subconjunctival injection of bevacizumab [42]. While most studies document a lesser effect of the anti-VEGF agents compared to MMC or 5-FU, these agents are not without risk and complications may still occur in a subset of patients.

## 7. Side Effects of Anti-VEGF Agents

Many adverse effects of anti-VEGF agents have been reported, including vitreous hemorrhage, lens injury, retinal detachment, central retinal artery occlusion, morphologic changes in corneal fibroblasts, and endophthalmitis. Elevated IOP, transient or sustained, is often a concern for patients with either ocular hypertension or pre-existing glaucoma [43].

In the initial treatment of NVG, additional intraocular volume from an injection frequently leads to worsened IOP elevation, necessitating anterior chamber paracentesis or aggressive medical management. This acute IOP elevation from anti-VEGF injection occurs in all eyes, but with a normally functioning outflow pathway, the majority of patients undergoing injections for conditions not resulting in NVG will return to a normal IOP in a matter of minutes [44]. Aside from this acute rise in IOP, persistent elevation in IOP does occur in a small subset of patients. Jalil et al. first reported a case in a patient with pre-existing ocular hypertension and receiving bevacizumab, but subsequent cases have been described in patients receiving each of the various agents and frequently with no prior history of ocular hypertension or glaucoma [45–48]. The reason for this phenomenon is unclear. There are reports of possible direct trabecular damage from contaminants, as well as some evidence suggesting that a low-lying inflammatory reaction leads to the effect [49, 50]. Another recent report suggested that the risk of requiring glaucoma surgery is elevated with accumulating numbers of injections, becoming more marked when patients require more than seven annual injections [51]. Regardless of the exact mechanism, closer observation of IOP is recommended as the frequency of injection increases.

## 8. Conclusion

The anti-VEGF agents have rapidly changed the management of many retinal conditions, including AMD, diabetic retinopathy, and vein occlusions. Their impact on surgical glaucoma is less clear. They have made the largest impact in the initial management of NVG. A single injection is often sufficient to induce regression of active neovascularization, which then facilitates initial surgical management and simplifies control of the underlying condition. Unfortunately, the long-term outcome of NVG remains poor with few studies showing a benefit to visual acuity or IOP with the adjunctive use of anti-VEGF medications. In traditional glaucoma surgeries, the use of anti-VEGF therapy has also met with limited success. Changes in bleb morphology after trabeculectomy or tube show the potential of these agents to influence the healing process; however, the long-term effects on IOP have yet to be realized.
